# Functional Objects in Urban Walking Environments and Pedestrian Trajectory Modelling

**DOI:** 10.3390/s23104882

**Published:** 2023-05-18

**Authors:** Andrew Kwok Fai Lui, Yin Hei Chan, Kevin Hung

**Affiliations:** School of Science and Technology, Hong Kong Metropolitan University, Hong Kong SAR, China; cyinhei@gmail.com (Y.H.C.); khung@hkmu.edu.hk (K.H.)

**Keywords:** pedestrian trajectory, pedestrian movement modelling, functional objects, deep learning, recurrent neural networks, urban environments

## Abstract

Functional objects are large and small physical entities installed in urban environments to offer specific functionalities to visitors, such as shops, escalators, and information kiosks. Instances of the novel notion are focal points of human activities and are significant in pedestrian movement. Pedestrian trajectory modelling in an urban scene is a challenging problem because of the complex patterns resulting from social interactions of the crowds and the diverse relation between pedestrians and functional objects. Many data-driven methods have been proposed to explain the complex movements in urban scenes. However, the methods considering functional objects in their formulation are rare. This study aims to reduce the knowledge gap by demonstrating the importance of pedestrian–object relations in the modelling task. The proposed modelling method, called pedestrian–object relation guided trajectory prediction (PORTP), uses a dual-layer architecture that includes a predictor of pedestrian–object relation and a series of relation-specific specialized pedestrian trajectory prediction models. The experiment findings indicate that the inclusion of pedestrian–object relation results in more accurate predictions. This study provides an empirical foundation for the novel notion and a strong baseline for future work on this topic.

## 1. Introduction

The increased urban population in the past few decades has prompted the emergence of large urban centers comprising shopping, entertainment, transportation, social, and other personal services under one roof [1,2]. Being in walking proximity to a wide range of functions is critical to an urban lifestyle [3,4]. The large and small objects installed to offer specific functionalities to visitors are called *functional objects*. Some functional objects are relevant to the principal reasons for the visit; for example, shops, fast-food stalls, and front offices of the authorities. Other objects, such as escalators, restrooms, information kiosks, and other amenities, are designed to enhance visitor experience or facilitate movement. The location of a function object is defined by its perimeter and orientation.

### 1.1. Modelling Functional Objects for Pedestrian Trajectory Prediction

Functional objects are focal points of pedestrian movements [5,6,7]. The microscopic movements in the surrounding area of a functional object are of particular interest. The trajectories can inform how individual users approach the object, how non-users move past the object and other aspects of pedestrian–object interaction. Understanding the diverse relation types between pedestrian movements and functional objects at the microscopic level and using it to predict the trajectory of individual pedestrians is helpful for many applications [8], including pedestrian facility engineering [9], public space development [2], evacuation [10], and technology-enhanced retail [11]. 

Microscopic pedestrian movement modelling has attracted much interest from computer science researchers. Pedestrian movement appears complex and may even be bordering on random [12]. However, it can be more fruitful to be considered as samples of heterogenous movement patterns resulting from the diverse types of pedestrian–object relations. In most situations, pedestrians have a functional object to pursue and can perceive and react to different objects in the environment [13]. The notion of relation has been proven useful in modelling pedestrian–pedestrian interactions. For example, Zhou et al. (2021) [14] modelled different reactive movements of a pair of pedestrians based on their relation. Sun et al. considered pedestrian–group relations in movement prediction [15]. Yucel et al. studied the connection between movement patterns and different types of relationships, including friends, colleagues, and couples [16]. Therefore, this paper aims to investigate the effectiveness of the notion of pedestrian–object relations in unlocking complex movements.

The notion of a functional object–pedestrian relation is novel in the context of pedestrian movement modelling. It resonates with the notions of location-of-interest (LOI), point-of-interest (POI), or sub-location used in relevant research works to represent an object of attraction [7,17]. The term POI is, however, more often referred to as a point of significance in a walking space rather than an object. For example, Ikeda et al. named the frequent points of making a turn as POIs [18]. Perhaps the most similar is the campus objects, or analogously dark matters, studied by Xie et al. [6], which exert an attractive or repulsive force on pedestrians. The functional object, on the other hand, can explain more than one distinctive movement pattern. It has a rich pedestrian–object relation model for organizing and classifying movement patterns.

### 1.2. Review of Relevant Research in Functional Objects

Many methods of pedestrian movement modelling have been proposed, and they can be divided into knowledge-driven and data-driven approaches [19]. The knowledge-driven methods exploit the rules and mechanisms from known physical, social, and personal features and are effective for modelling simple behaviors. These models have a strong assumption of the movement pattern and use a few hand-engineered features to describe the movement. For example, the acceleration and velocity features can adequately describe slowing down and stopping pedestrians [20]. Other examples include Kalman filters [21], the hidden Markov model [22], the collision avoidance models [23], and the social force model [24]. However, these models can become grossly inadequate when transferred to a novel scenario. To apply the knowledge-driven approach in the pedestrian–object relation framework, a specialized hand-crafted model is required for every relation type. Yue et al., Li et al., and Sun et al. [25,26,27] have illustrated the effort required to develop such a model. 

The data-driven methods learn the rules and features from trajectory data. These methods usually consider the trajectory of movement as a time series and use the recurrent neural networks (RNN) and their variants to learn to predict the next locations from the previous locations [28]. The data-driven approach has significantly eased the modelling of specific scenarios. The approach can produce specialized models through filtering or subsampling a training trajectory dataset or further training a general model with training data of the desired context. For example, Xue et al. divided the training data into route classes and used the same training architecture to obtain a route class movement model for every route class [29]. The specialized models were found to outperform the general movement model.

### 1.3. Pedestrian-Object Relation Guided Trajectory Prediction (PORTP)

An approach to develop a data-driven modelling method for the pedestrian–object relation framework is to inform the training architecture of the relation type associated with every trajectory sample. Before model training, the samples in the training dataset are augmented with the relation variables using unsupervised learning. The resulting model has the required context to encode and decode movement patterns according to the relation type. While this method can learn complex behaviors, the model training may be hindered if some relation types are under-represented in the training data. For example, if the number of users of a functional object is substantially less than the number of non-users, then the resulting training sample and the learned features in the model will be heavily biased.

The proposed modelling method, pedestrian–object relation guided trajectory prediction (PORTP), resolves the problem by defining a range of specialized trajectory prediction (STP) models for each pedestrian–object relation. Each model is specialized for predicting movements associated with a functional object, an intent class, and a mode of the relation. In the prototype implementation, the possible intents include user and non-user, and the possible modes include cruising, approaching, and being engaged. For example, there will be a model for explaining the users of an escalator in the approaching mode and another model for explaining the non-users near a ticket office in the cruising mode. The method assumes the prior collection of a training dataset of pedestrian trajectories. For training the specialized models, the dataset is divided into a group of specialized datasets based on unsupervised learning. The proposed method defines a classifier for predicting the relation type of the trajectory of a pedestrian and selects the STP models accordingly. Hence, the resulting architecture is dual level, consisting of a set of STP models on one level (including a general model as a fallback) and a relation classifier on another level. 

### 1.4. Contribution and Structure of the Paper

The main contribution of this paper is the dual-level architecture for modelling individual pedestrian movement while taking into account the relation type with functional objects. Based on the performance evaluation that will be described in this paper, the significance of functional objects in pedestrian movement modelling is evidenced, and the trajectory prediction accuracy of the users of functional objects have been found improved over the modelling architectures that do not consider functional objects. The training and evaluation of the method were set in the context of three functional objects selected from a multi-functional center in Osaka. 

The next section gives a thorough analysis on the pedestrian–object relation notion by connecting the elements to the literature. It also describes the deep learning data-driven techniques used to develop our proposed architecture. The paper then presents the architecture, notes on the prototype implementation, and the training and evaluation plan. It is followed by a report on the performance evaluation of the DTP models, the relation classifier, and the overall movement models of the three scenarios. Based on the findings from the evaluation, some concluding remarks, including suggestions for future research, are finally made.

## 2. Characteristics of Functional Objects

**Figure 1:**
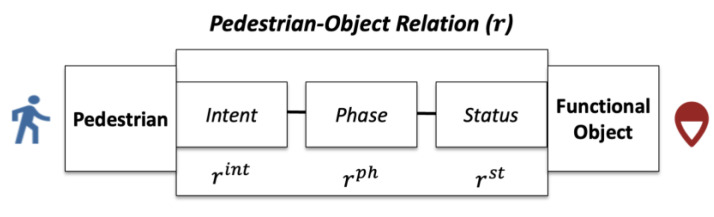
The pedestrian–object relation between a pedestrian and a functional object.

The pedestrian–object relation is defined as a 3-tuple of pedestrian-intent, object-status, and the phase of the relation, as shown in Figure 1. Each of the three aspects can find support in the literature. The pedestrian-intent aspect specifies whether the pedestrian intends to be a user of the object. Hidaka et al. [17], Xie et al. [6], and Kielar and Borrmann [7] studied the connection between the intent and the target location of movement. The object-status aspect includes the dynamic features of the function object. Examples are the dynamic signals of a traffic light [30] and the opening status of a door [31]. Finally, the phase of the relation is reserved for explaining the changing movement patterns that may emerge in the interaction between pedestrians and objects. For example, Feliciani et al. identified the changing movement patterns of pedestrians during a typical interaction with a crosswalk, namely, moving toward a crosswalk, preparing to step onto the crosswalk, and then to walking on the crosswalk [32].

The proposed definition provides a comprehensive framework for the microscopic modelling of pedestrian movement. Each relation type, a particular combination of the three relation variables, corresponds to specific movement patterns. The modelling problem can be simplified into modelling a significantly more homogeneous pedestrian movement sample for every relation type. 

### 2.1. Pedestrian and Functional Object Relation

The functional object is significant to pedestrian movement modelling. It specifies the locations where users must reach to engage its function. The engaged locations are often outside the perimeter of the object. For example, information kiosks and ticket offices engage users at locations on the other side of the counter. The engaged locations may be derived from the functional object’s location as external knowledge, but they are often extracted from movement data empirically [33]. Hidaka et al. divided a recreational park into grids and considered the ones most stayed at by pedestrians as the POI [17]. Bennewitz et al. considered the locations where many people stopped and stayed at for some time as intermediate destinations [34]. However, the condition for detecting the engaged locations is dependent on the functional object. For example, it is a stop for an information kiosk, but a constant velocity for an escalator.

Several relevant research works utilized the pedestrian–object relation in their formulations. However, these prior relation models were single-aspect, compared to the three-aspect relation defined in this work. 

#### 2.1.1. The Pedestrian-Intent Aspect

The pedestrian-intent aspect indicates how likely the functional object is to be the destination of the pedestrian. The value can be a probability, the rank among the objects, or a binary variable (i.e., user or non-user). Many researchers have already studied this aspect as the topic of destination choice modelling [6,7,17]. 

Destination choice modelling involves choice set generation, which determines the candidates of functional objects [35]. The choice set can be determined by external knowledge, empirical analysis, or a combination of the two. For example, the list of functional objects is prior knowledge. However, when there are many functional objects and the problem becomes too complex, the more distant objects may be ruled out. A method to reduce the size of the list is to divide the scene into zones of analysis [36]. Another method is to select popular functional objects using unsupervised learning of pedestrian trajectories [6,18].

#### 2.1.2. The Object-Status Aspect

The object-status aspect indicates if the object has a significant change that may affect its relation to all the pedestrians. It is rarely studied in the context of urban environments. One rare example is the status of automatic doors [31], which changes the accessibility of a walking space. Other potential statuses include engaged status and broken status. Each functional object may have several statuses, and their values are usually linked to an external knowledge source.

#### 2.1.3. The Phase-of-Relation Aspect

**Figure 2:**
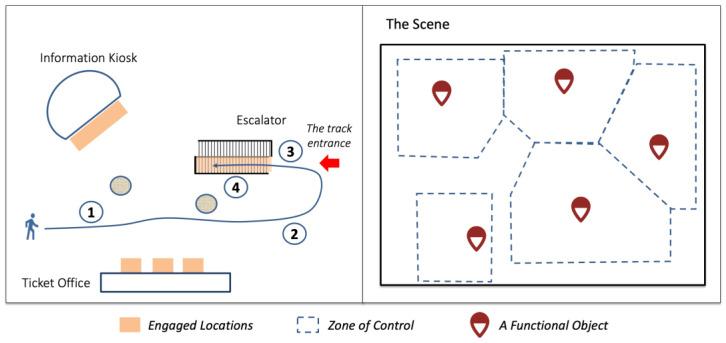
On the left is an illustration of 3 functional objects, the engaged locations specified for these objects, and a pedestrian, who is a user of the escalator, moving to the escalator in 4 phases. On the right is an illustration of how the scene is divided into modelling zones, each occupied by one functional object.

The phase-of-relation aspect models the phase change that may happen during the interaction between a pedestrian and a functional object. The phase change is associated with distinctive movement patterns. For example, an escalator user starting from some distance from the destination goes through several phase transitions: (1) cruises in a minimal-effort manner until the escalator is visible; (2) moves to the side where the track entrance is located; (3) adjusts the direction and speed to align with the track; (4) steps on the track. Figure 2 Illustrates the phase transition In a relation between a pedestrian and the escalator. Some studies have noted phase changes in pedestrian movement. For example, Hahm et al. noted a non-user of a shop who walks in a usual manner but then suddenly slows down due to the attraction of the shop window [37]. Likewise, Feliciani et al. identified several movement patterns in the phases before and during walking across a crosswalk [32]. 

### 2.2. Pedestrian-Object Relation and Microscopic Movement Models

As microscopic pedestrian movement in the urban environment is inherently complex, many researchers have proposed methods to ease the modelling task. Some effective methods are based on considering the movement at two or more levels of abstraction. For example, Hoogendoorn et al. proposed a three-level framework consisting of a strategic level (i.e., activity planning), tactical level (i.e., destination choice and route choice), and operational level (i.e., inertia, interaction with the environment and obstacles, and interaction with other pedestrians) [38]. Hoogendoorn et al. also introduced a two-level framework combining global route choice and local route choice to recover the latent self-organized movement patterns [39]. Hidaka et al. used the top layer to model the Intent on POIs and the bottom layer to generate the trajectories under the constraint of the destination in a two-layer architecture [17]. 

The multi-layer approach has several advantages. First, the model parameters of different abstraction levels can be optimized independently, which reduces computation effort and improves performance [39]. Second, the multi-layer modular architecture can support a mix-and-match of different multi-model combinations. Third, the multi-layer architecture can facilitate the division and specialization of the model, with an upper level as a classifier of cases and the lower level as a provider of STP models for every class of cases [29,40]. 

### 2.3. Route-Class Modelling (PoPPL)

The prediction of pedestrian paths by the LSTM (PoPPL) method is an advanced example of the multi-layer architecture [28]. The top layer is a route class classifier of trajectories, and the bottom layer comprises STP models for the route classes. The route class is a pairing of origin and destination, each of which may be regarded as a POI or functional object. The method assumes that the trajectories of the same route class are homogeneous—pedestrians moving between the same origin–destination pair follow similar paths. 

The classifier and the specialized models are all data-driven and trained using a deep neural network architecture based on long short-term memory (LSTM). LSTM is a variant of RNN that can learn the latent long-term features in the trajectories. The same encoder–decoder architecture was designed to train all the STP models. The training data for each STP model were the extracted trajectories of the corresponding route class.

### 2.4. Destination-Driven Modelling (DDPTP)

The destination-driven pedestrian trajectory prediction (DDPTP) is also a dual-layer architecture [41]. A significant difference from PoPPL is the use of the destination class rather than the route class. The destination class represents the intent of an LOI and, therefore, a step toward the pedestrian–object relation. An improved method based on DDPTP introduced three phases of relation as a pedestrian is moving near a functional object [40]. The three phases are *engaged*, *approaching*, and *passing-by*, each mapped to an STP model for the functional object. The method divides the scene into zones, each of which contains a functional object, and, as a consequence, splits the classification task into classifiers of every zone. The improvements increased the prediction accuracy compared to PoPPL and DDPTP.

### 2.5. Feature Learning of Pedestrian Movement Patterns

The RNN is a well-proven deep learning architecture for learning features in sequential data. It keeps an internal state for analyzing and encoding the input sequences into implicit features. Its superior variants, the LSTM and the gated recurrent units (GRU), are used in practice due to the ability to analyze long sequences. For example, in pedestrian trajectory prediction tasks, the extracted movement features can be coupled to a classifier for intent or relation classification. The features can also be coupled to another LSTM or GRU cell for the generation of predicted trajectories, and this configuration is known as the encoder–decoder architecture. The role of the encoder is to learn high-level features from the input features.

The kinds of high-level features that may be extracted depending on the input features. The time series of the absolute locations of pedestrians (based on the reference frame of the environment) can facilitate the learning of the walking scene [42], including the accessible locations and estimated goals [43]. The velocities of pedestrians can inform the movement patterns, such as stopped or slow movement [28]. Some relevant proposed methods considered other objects in the environment, such as pedestrians [44], robots [45], and functional objects. The use of relative locations and velocities to the nearby pedestrians is the basis of much research on social interaction in trajectory prediction. It is reasonable to expect that the same technique is also effective in modeling the interactions with robots and functional objects. 

Reference values are essential for the semantic interpretation of many features such as speed and direction. For example, the high-level feature of cruising requires a reference of the usual speed of all or a subset of pedestrians. In addition, location-dependent reference values, often represented as a heatmap, can inform interactions between pedestrians and the environment [46]. Examples of heatmaps include the presence of stationary pedestrians [47] and significant waypoints (i.e., where pedestrians often change direction) [48]. In particular, the locations where many pedestrians changed their movement patterns (e.g., suddenly stop, slow-down, or make a turn) may also be the place a function object becoming visible [49] or attractive [50].

## 3. Method

The interactions between functional objects and pedestrians are complex, making the explanation of pedestrian trajectories challenging a challenging task. Based on the above discussion, the intent of pedestrians, the status of functional objects, and the phase of their interactions can help organize the trajectories into more homogeneous sets for modelling. The result is PORTP, a pedestrian–object relation framework, that can guide more effective data-driven modelling of pedestrian movements. Like PoPPL and DDPTP, PORTP is a dual-layer architecture accommodating an array of STP models. PORTP is a generic method for modelling different types of functional objects through the pedestrian–object relation framework.

### 3.1. Overview of the Problem

The problem is defined as follows. Given an observed movement sequence of a pedestrian ending at time τ called $s^{\tau .z}$ where $s^{\tau ,z}=\left[\langle x_{\tau -z+1},y_{\tau -z+1}\rangle ,\cdots ,\langle x_{\tau },y_{\tau }\rangle \right]$ consists of z observations of (x,y) positions from time τ−z+1 to τ, the task is to predict the future z positions ${\hat{s}}^{\tau +z,z}=\left[\langle {\hat{x}}_{\tau +1},{\hat{y}}_{\tau +1}\rangle ,\cdots ,\langle {\hat{x}}_{\tau +z},{\hat{y}}_{\tau +z}\rangle \right]$. The rudimentary deep learning method of building the pedestrian trajectory prediction model $\psi \left(\cdot \right):\;s^{\tau ,z}\to {\hat{s}}^{\tau +z,z}$ involves feature learning from S, which is a set of observed movement sequences of length 2z extracted from the raw dataset. 

In the PORTP method, the universal model ψ(·) is supplemented by the STP models $\langle {\tilde{\psi }}_{1},\;{\tilde{\psi }}_{2},\;\cdots ,\;{\tilde{\psi }}_{kmax}\rangle$ where $k_{max}$ is the number of instances in the pedestrian–object relation set. The same index from $\langle 1,\;\cdots ,k_{max}\rangle$ uniquely identifies the relation type and its respective STP models. The pedestrian–object relation r is a 3-tuple $r=\left(r^{int},r^{st},r^{ph}\right)$, where $r^{int},r^{st}$, and $r^{ph}$ are the pedestrian-intent, object-status, and phase-of-relation aspects, respectively. The relation set is pre-determined based on external knowledge. PORTP uses the pedestrian–object relation tracker $\gamma \left(\cdot \right):\;s^{\tau ,z}\to r$ to determine the relation type of a pedestrian movement sequence, which informs the selection of the STP model. 

**Figure 3:**
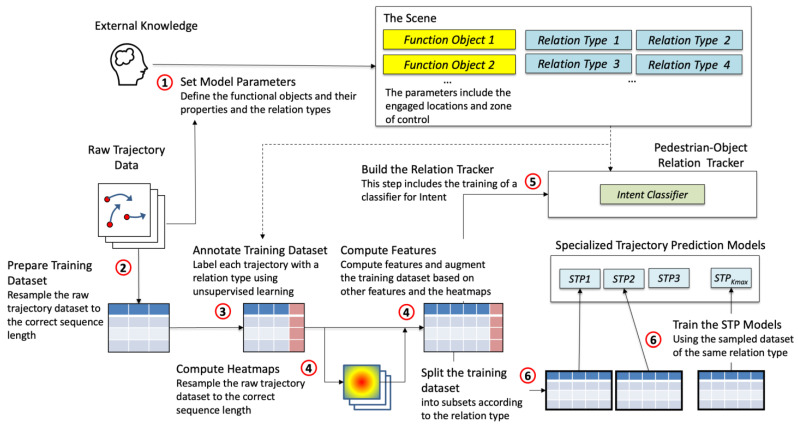
The PORTP method illustrated with the tasks in every step. The numbers in red circles indicate the steps of the method. The arrows with a solid line indicates the dependency of the steps and the arrows with a broken line represent information flow.

Figure 3 shows the proposed method in a step-by-step manner. It is assumed that a human such as an analyst has defined the modelling areas in an urban environment, obtained relevant information on the functional objects in the areas, and collected a trajectory dataset. The analyst then specifies the $M_{max}$ functional objects $\langle fo_{1},\;fo_{2},\;\cdots ,\;fo_{Mmax}\rangle$ and the relation types for every functional object, including their parameters. The engaged location of a functional object may be calibrated using the trajectory dataset and unsupervised learning (e.g., clustering of the locations where pedestrians stopped moving).

In the next step, the training dataset is prepared by cleansing and re-sampling the raw trajectory dataset. Then, the samples are annotated with the ground-truth relation type using a combination of rules and cluster analysis. After this, the feature set of the training dataset is expanded. Several reference values and heatmaps are prepared for the computing of four sets of input features.

The pedestrian–object relation tracker is essentially rule-based, except the intent classifier is based on deep neural networks. The classifier is trained on the expanded training dataset using supervised learning. Finally, the training dataset is split according to the relation type and the functional object, and each subset is used to train a STP model.

### 3.2. Specify the Model Parameters

Every functional object $fo_{m}$ is defined with its location $Z^{F}$, the engaged locations $Z^{N}$ and the zone of control $Z^{C}$, which are sets of (x,y) positions. The engaged locations may be calibrated. Given the raw trajectory dataset and a predicate for the engaged condition, for every position near the functional object, the number of trajectories that match the condition can be tallied. The engaged locations can be re-defined using the more frequently visited positions. The zones of control of function objects are assumed to be non-overlapped.

A set of relation types is also defined for every function object. There is no limit on the classes of intents, phases, and statuses between pedestrians and a functional object. However, of the most basic definition, the intent is either *user* or *non-user*, and the phase is one of the *engaged*, *approaching*, and *passing-by*, and the status is simply undefined.

### 3.3. Prepare the Training Dataset and Enrich the Input Features

The intent classifier of the relation tracker γ(·) and the STP models $\langle {\tilde{\psi }}_{1},\;{\tilde{\psi }}_{2},\;\cdots ,\;{\tilde{\psi }}_{kmax}\rangle$ are data-driven models. The model architecture requires a time series of a pedestrian movement feature set of length 2z as the format of the input training sample. In order to learn strong features related to the interaction between pedestrians and functional objects, the input feature set includes scene-referenced movement features, object-referenced movement features, pedestrian interaction features, and location-dependent reference values. 

PORTP assumes that the source dataset contains a set of trajectory samples of sufficient size, of which each sample is a time series of absolute positions of a pedestrian of an arbitrary length. The training dataset is prepared by, first, subsampling every trajectory sample into overlapping sequences of length  2z, and second, enriching the input feature set as described below.

#### 3.3.1. The Scene-Referenced Movement Features

These features contain a sequence of positions $s^{\tau ,z}$ and a sequence of velocities $v^{\tau ,z}$ derived from $s^{\tau ,z}$. The scene is the frame of reference, and the measurements are considered as absolute.

#### 3.3.2. The Object-Referenced Movement Features

These features contain a sequence of positions and a sequence of velocities relative to the functional object of the zone of control. The function location centric function π(·) maps the scene-referenced positions to the object-referenced positions $r^{\tau ,z}$ using the location of the functional object ${\langle x,y\rangle }^{fo}$.

(1) $$r^{\tau ,z}=\pi \left(s^{\tau ,z}\;,\;{\langle x,y\rangle }^{fo}\right)$$

#### 3.3.3. The Pedestrian Interaction Features

These features represent the number of nearby pedestrians. The vicinity of the agent is divided into an occupancy map consisting of distance-dependent zones. For the agent i at time step τ, the neighborhood $N^{\tau }$ is extracted as follows.

(2) $$N^{\tau }=\langle {\langle x_{\tau },y_{\tau }\rangle }_{a}\;|\;\forall a:\;\;a\neq i\;and\;{\langle x_{\tau },y_{\tau }\rangle }_{a}\subset occupancy\;map\;of\;i\rangle$$

The pedestrian interaction function $O^{C}\left(\cdot \right)$ computes the pedestrian interaction features as follows.

(3) $$O^{C}\left(s_{i}^{\tau ,z},\;N^{\tau }\right)$$

**Figure 4:**
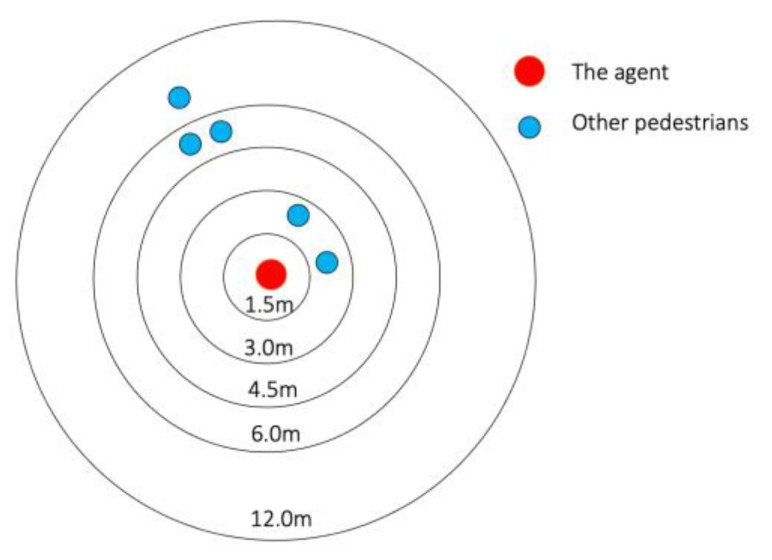
The occupancy map. In this example, the feature vector is [0, 2, 0, 2, 1] with each element representing the number of other pedestrians in the 5 zones from the nearest to the furthest.

Figure 4 below illustrates the occupancy map and an illustration of the features with a scenario.

#### 3.3.4. The Location-Dependent Reference Values

These features represent the characteristics of the agent’s current position. To compute these features, the following five heatmaps, which are functions of location in the scene, are first calculated.

- ${ℳ}^{Cr}$: the mean speed of pedestrians at the location (i.e., the cruising speed).
- ${ℳ}^{Stop}$: the probability of a pedestrian stopped at the location.
- ${ℳ}^{Slow}$: the probability of a pedestrian slowed down at the location.
- ${ℳ}^{Turn}$: the probability of a pedestrian making a turn of 90 degrees of more.
- ${ℳ}^{User}$: the probability of a pedestrian being the user of the functional object in the same zone.

The five heatmaps are calculated from the trajectory dataset and the locations of the function objects. Finally, the features at the agent’s position can be looked up from the heatmaps.

### 3.4. Build the Pedestrian-Object Relation Tracker

The purpose of the tracker is to determine and track the relation $pr_{i,m}$ between a pedestrian i and functional object m. It is called a relation tracker because the current relation is one of the input parameters of the tracker. The other input parameters include the trajectory $s^{\tau .z}$, the location and the zones of the related functional object $\langle Z^{C},Z^{N},Z^{F}\rangle$, and the intent classifier ω(·). Every pedestrian is related to at most one functional object, which can be determined by the zone of control that the pedestrian is located.

The tracking algorithm is rule-based, and an example based on the basic configuration of the relation framework is described in Algorithm 1.

(4) $$pr_{i,m}{}^{new}=\gamma \left(s_{i}^{\tau ,z},\;pr_{i,m},\langle Z^{C},Z^{N},Z^{F}\rangle ,\;\omega \left(\cdot \right)\;\right)$$

**Algorithm 1** Tracking of pedestrian–object relation in a zone of control**Input:** The trajectory $s^{\tau .z}$ with the latest position $\langle x_{\tau },y_{\tau }\rangle$ of a pedestrian agent i, the current relation $pr_{i,m}$ with respect to the function object $fo_{m}$, the location and the zones of the object $\langle Z^{C},Z^{N},Z^{F}\rangle$, and ω(·) is the intent classifier.
**Output:** The updated behavior class $pr_{i,m}{}^{new}$1 2 3 4 5 6 7 8 9 10 11 12 13 14 15 16 17$i\;\langle x_{\tau },y_{\tau }\rangle \;\notin \left(Z_{m}^{C}\cup Z_{m}^{E}\right)$:   *# pedestrian left the ZOC of the object*  return None if $pr_{i,m}.phase==$ *Unknown*:  $pr_{i,m}.phase\leftarrow$ *passing-by* if $pr_{i,m}.intent==user$: if $pr_{i,m}.phase==$ *approaching* and $\langle x_{\tau },y_{\tau }\rangle \;\in \;Z_{m}^{N}$: * # entered engaged locations*  $pr_{i,m}.phase\leftarrow$ *engaged* $\mathrm{elif}\;pr_{i,m}.phase==$ *engaged* and $\langle x_{\tau },y_{\tau }\rangle \;\notin \;Z_{m}^{N}$: * # left engaged locations*  $pr_{i,m}.phase\leftarrow$ *passing-by* else:  $pr_{i,m}.intent\;\leftarrow \;\omega {\left(s^{\tau .z}\right)}_{m}\;$    *# changed intent*  $pr_{i,m}.phase\leftarrow$ *passing-by* $\mathrm{elif}\;pr_{i,m}.intent==nonuser$: $pr_{i,m}.intent\;\leftarrow \;\omega {\left(s^{\tau .z}\right)}_{m}\;$ if: $pr_{i,m}.phase==user:$  $pr_{i,m}.phase\leftarrow$ *approaching* return $pr_{i,m}$

#### The Intent Classifier

While the related function object can be determined by the zone of control, and the phase can be determined by rules based on the engaged locations and other conditions, the intent, has to be estimated from pedestrian movement. 

The network structure is specified by the following equations, in which $pd^{intent}$ is the intent class’s probability distribution, FC is a fully connected neural network, $FC^{Softmax}$ is one with a *softmax* layer at the output, and $\langle f^{SPM},f^{CR},\;f_{ℒ}^{FPM},f_{ℒ}^{LDR},f^{PI}\rangle$ are the feature sets obtained from the pedestrian behavior encoder and the functional location induced behavior encoder.

(5) $$\omega :\;s^{\tau .z}\to pd^{intent}=FC^{Softmax}\left(FC\left(f^{SPM}\;⊕f^{CR}\;⊕f^{FPM}\;⊕f^{LDR}\;⊕f^{PI}\;\right)\right)$$

(6) $$f^{SPM}=GRU^{PB}\left(e^{t,z}\right)\;where\;e^{t,z}=\theta \left(s^{t,z},v^{t,z}\right)\;$$

(7) $$f^{CR}=FC\left(GRU^{CB}\left({ℳ}^{Cr}\left(s^{\tau ,z}\;\right)\right)\;⊕\;GRU^{CR}\left(v_{t}^{\tau ,z}\right)\right)\;$$

(8) $$f_{m}^{FPM}=FC\left(GRU^{FPM}\left(r^{\tau ,z}\right)\;\right)\;where\;r^{\tau ,z}=\pi \left(s^{\tau ,z}\;,\;Z^{F}\;\right)\;$$

(9) $$f_{ℒ}^{LDR}=\langle {ℳ}^{Stop}\left(\langle x_{\tau },y_{\tau }\rangle \right),{ℳ}^{Slow}\left(\langle x_{\tau },y_{\tau }\rangle \right),\;{ℳ}^{Turn}\left(\langle x_{\tau },y_{\tau }\rangle \right)\rangle$$

(10) $$f_{ℒ}^{PI}=FC\left(GRU^{PI}\left(O^{C}\left(s_{i}^{\tau ,z},\;N^{\tau }\right)\right)\;\right),\;\mathrm{where}\;\;N^{\tau }=\langle {\langle x_{\tau },y_{\tau }\rangle }_{a}\;|\;\forall a:\;\;a\neq i\;and\;{\langle x_{\tau },y_{\tau }\rangle }_{a}\subset occupancy\;map\;of\;i\rangle$$

**Figure 5:**
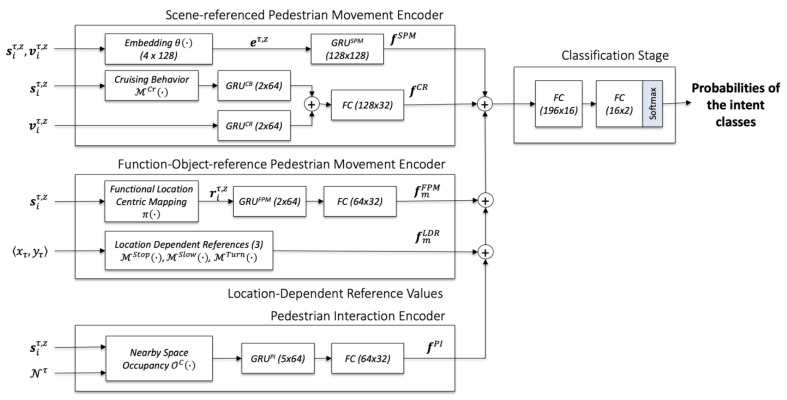
The deep neural network architecture for training the intent classifier.

The deep neural network architecture for the intent classifier is illustrated in Figure 5. The target output is the probability distribution of the intent classes <*user*, *non-user*>. There are two choices of the ground truth. The first one is the pedestrian ground truth, obtained from the annotated relation as described in Section 3.1. The second one is the locational ground truth, which is looked-up from the pre-computed heatmap ${ℳ}^{User}$.

### 3.5. Train the Specialized Trajectory Prediction Models

The generic STP models is based on the encoder–decoder architecture. The encoder GRU uses the network structure described in the following equations to learn movement features for a relation instance, where $e^{t,z}$ is an embedded vector of the input movement sequence computed by θ(·), $f_{ℒ}^{LDR}$ is the set of stopping, slowing down, and turning features as described before, and $h_{\tau }$ and $h_{\tau -1}$ are the hidden states of the current and previous training step.

(11) $$\;h_{\tau }=GRU^{ENC}\left(e^{t,z}\;⊕\;\left(FC\left(e^{t,z}\;⊕\;f_{ℒ}^{LDB\prime }\right)\right),\;h_{\tau -1}\right)\;\;$$

(12) $$e^{t,z}=\theta \left(s^{t,z},v^{t,z}\right)\;$$

(13) $$f_{ℒ}^{LDR}=\langle {ℳ}^{Stop}\left(\langle x_{\tau },y_{\tau }\rangle \right),{ℳ}^{Slow}\left(\langle x_{\tau },y_{\tau }\rangle \right),\;{ℳ}^{Turn}\left(\langle x_{\tau },y_{\tau }\rangle \right)\rangle$$

The final hidden state at time τ is passed to the decoder side for the generation of future movement based on the current position. Assuming that the network training is completed, the next position is generated as follows.

(14) $${\tilde{\psi }}_{k}:\;s^{\tau ,z}\to \langle x_{\tau +1},y_{\tau +1}\rangle =FC\left(GRU^{DEC}\left(\langle x_{\tau },y_{\tau }\rangle ,\;h_{\tau }\right)\right)\;\;$$

(15) $$h_{\tau +1}=GRU^{DEC}\left(\langle x_{\tau },y_{\tau }\rangle ,\;h_{\tau }\right)$$

**Figure 6:**
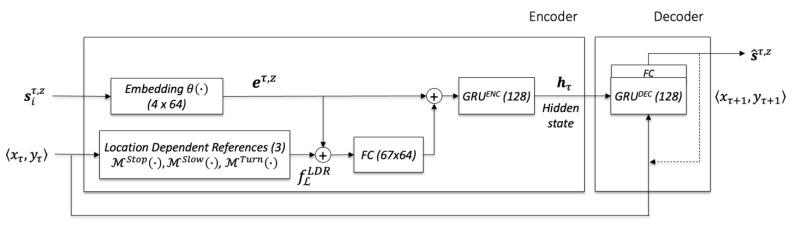
The generic deep neural network architecture for training the STP models.

Figure 6 shows a graphical representation of the deep learning architecture for training generic STP models.

To save the computational requirement of the data-driven approach, three alternative hand-crafted STP models are used for simple movement patterns, including *waiting*, *moving slowly* and *steadily*, and wandering slowly in a small area. These STP models are mainly used for the engaged phase. For example, the *waiting* model can explain an engaged pedestrian at an information kiosk.

## 4. Experiments and Results

PORTP has been implemented using *python 3* with *pytorch*. Refer to the above figures for the dimension of the two data-driven models. The optimizer is *RMSprop*, the learning rate is 0.001, the dropout rate is 0.5, and the number of training epochs is 1000. The GTX-3090 GPU has been used to support the model training. 

### 4.1. Dataset

The experiment is based on the trajectory dataset collected by 3D range sensors installed in and around the Asia and Pacific Trade Center (ATC) in Osaka, Japan [51]. The ATC is a shopping center, transportation hub, and conference center rolled into one. The scene of the dataset is a walkway connecting a railway station to a large forum; the full dimension measures over 140 m × 60 m. 

The original dataset contains 92 days’ worth of daily observations of over 10 h each day, so a subset has been selected for the experiment. The data between 24 October 2012 and 28 October 2012, three functional objects, and their zones of control are included in the experiment, including the ticket office and the escalator about the forum on the western end and the information kiosk on the eastern end. After the resampling, the trajectory length 2z=24. 

**Figure 7:**
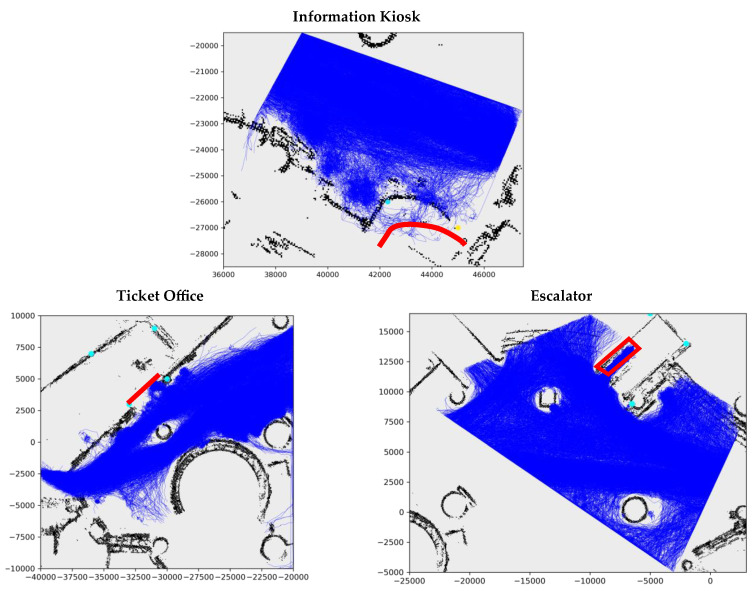
The scenes of the three selected functional objects in the ATC, and the trajectory plots of all the pedestrians included in the training sample (in blue). The locations of the functional objects are also indicated (in red). The unit of both axes is millimeters.

Figure 7 shows the extracted scenes near the three selected functional objects and the training trajectory samples for each object. The locations of the functional objects are also indicated. Much of the walking space is occupied by the trajectory samples. Most trajectories do not touch the objects, suggesting that most samples are non-users (consistent with Table 1). In the scenes of the information kiosk and escalator, the users’ trajectories are more visible as they enter the regions in red (the engaged locations). However, the users and non-users are harder to distinguish in the scene of the ticket office. 

**Figure 8:**
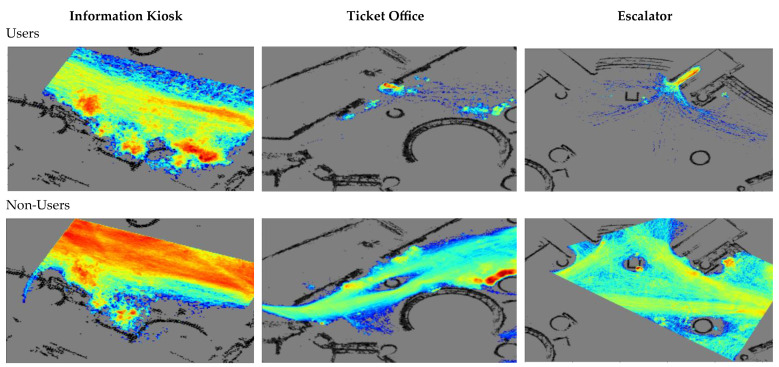
The density of trajectories of users ((**top**) row) and non-users ((**bottom**) row) of the three functional objects. Red indicates high density, and blue indicates low density. The two rows of the color-coded visualization are on different scales.

Figure 8 illustrates the densities of trajectories of the users and the non-users of the three functional objects. For example, most non-users moved along the walkway bordering the kiosk in the information kiosk scene. The users were mainly found next to the information kiosk, along the near side of the walkway, and where the brochures were displayed (on the left). The patterns of the users of the ticket office and escalators are also clearly visible. 

**Figure 9:**
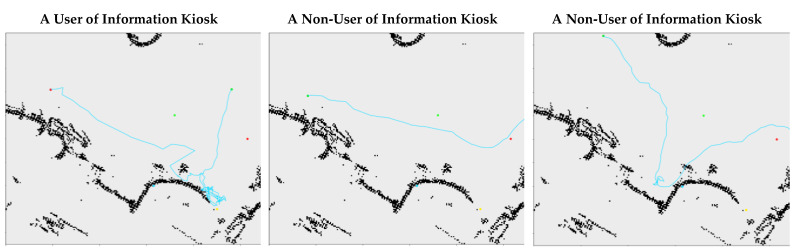
The trajectory of a user of the information kiosk is on the left, and the trajectories of two non-users are in the center and on the right. All of them move from right to left along the cyan line.

The differentiation of movement patterns between users and non-users can be minimal. Figure 9 demonstrates this with selected samples from the dataset of the information kiosk. Their trajectories share similarities, but they were one user and two non-users.

### 4.2. Evaluation and Metrics

The measurements used in the experiments include the average displacement error (ADE) and the final displacement error (FDE). The former computes the mean error over the entire predicted trajectory, and the latter measures the discrepancy at the final step. The following computes the ADE and the FDE for one predicted trajectory.

(16) $$\mathrm{ADE}=\frac{1}{z}\overset{2z}{\underset{t=z+1}{\sum }}∥s^{\tau +z.z}{}_{t}\;-\;{\hat{s}}^{\tau +z,z}{}_{t}∥$$

(17) $$\mathrm{FDE}=∥s^{\tau +z.z}{}_{2z}\;-\;{\hat{s}}^{\tau +z,z}{}_{2z}∥$$

The performance of PORTP is evaluated using five-fold stratified cross-validation. For the intent classifiers, the *user* class is augmented to overcome the representation problem of a small class.

### 4.3. Quantitative Evaluation 

The functional object defined in this paper is a new notion in the research area of pedestrian trajectory prediction. PORTP is unique as a method that analyzes the movement patterns resulting from the interaction between a pedestrian and a functional object. In evaluating PORTP, PoPPL [32] is chosen as the baseline for the following reasons. First, both PORTP and PoPPL address the role of the environment, specifically LOIs, in microscopic pedestrian movement. In addition, both operate on the microscopic level in a continuous movement space. Finally, PoPPL has achieved state-of-the-art performance and even significantly outperformed the models that have taken social interactions into account.

The GRU-only model is based on encoder–decoder architecture, one of the baseline models selected in many studies in pedestrian trajectory modelling. It represents the scenario that the functional object is not part of the formulation. 

Table 2 summarizes the ADE and FDE of testing the three models against three subsets of test cases, including the whole original set, only the users, and the pedestrians near the function object. PORTP gave the best performance in predicting users of functional objects, outperforming the baselines significantly in all the functional objects. Among the three functional objects, the escalator scene brought out the best performance of PORTP, which gave better accuracies than the baselines. In the scene of the information kiosk, PORTP performed marginally better in the all-test-cases scenario. However, PORTP did not have any advantage over the rivals in the scene of the ticket office (except the user scenario). 

The next part of the evaluation is on the effectiveness of the principal components in PORTP. Table 3 shows the prediction accuracy of the intent classifier. P represents the user class, and N represents the non-user class. The baseline models include the PoPPL classifier, of which pedestrian movement is the only input feature. With the input features relevant to the function objects, PORTP outperformed PoPPL significantly in the scenes of all three functional objects.

Table 4 compares two different ground-truth specifications used to train the intent classifier. Again, the default of using locational ground truth gave significantly better prediction accuracy than using the pedestrian ground truth.

Table 5 compares the accuracy of the intent classifier in three different scenarios based on the pedestrians’ distance from the functional objects. The general observed trend is that the prediction accuracy is higher when closer, but the changes are not the same among the objects. The classifier for the escalator displayed the largest variations, meaning that few learnable features could differentiate between users and non-users when they are far away from the object.

The evaluation of the performance of the STP models is shown in Table 6. The PORTP models, augmented with the location dependent reference values, generally gave better ADE and FDE than the GRU encoder–decoder models. The exception scenario is non-user, passing-by model for the ticket office. The probability of pedestrians stopping, slowing down, and turning at a location helped improve the trajectory prediction near the information kiosk and the escalator.

### 4.4. Qualitative Evaluation 

**Figure 10:**
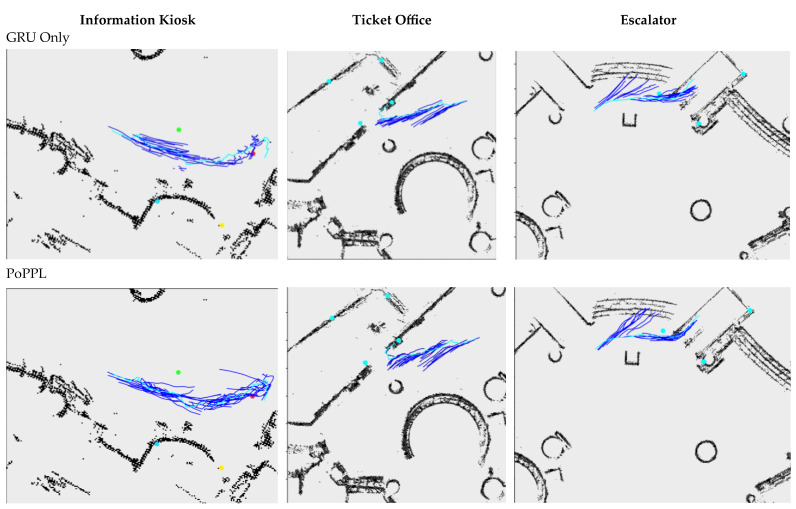

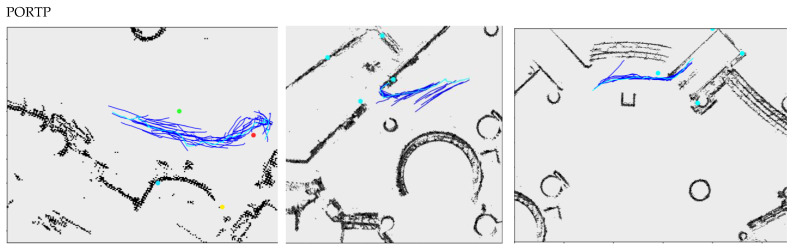
The comparison of the observed (ground-truth) trajectories (in cyan) and the predicted trajectories (in blue) of the users of the three functional objects. The agent moved from right to left in the information kiosk and ticket office scenarios. The agent moved from left to right in the escalator scene. All the predicted trajectories originated from the same observed trajectory, but at subsequent locations.

Examples of predicted trajectories of selected agents who are users of functional objects are shown in Figure 10. PORTP did well in the scenes of the information kiosk and escalator. The kinds of characteristic movements and the locations of their appearance are consistent with the ground truth.

The GRU-Only model’s predictions are momentum-based, and therefore most predicted trajectories are lines of tangents emerging from the observed trajectories. This behavior is evident in the escalator scene (as shown in the top-right plot). The PoPPL’s predictions include some characteristic movements, but the locations of their emergence are less accurate than PORTP.

In the scene of the ticket office, all the models did poorly when the pedestrian was further away from the object. The predicted trajectories were more consistent with those of the non-users. The error is due to the misclassification of the intent. 

## 5. Discussion

An important finding from the results of the experiments is the significance of functional objects to pedestrian movements. In general, the models that consider functional objects can explain the nearby trajectories better than those that do not include functional objects in the formulation. Additionally, the better models are those trained with more homogeneous training datasets and utilized features engineered from a functional object.

The differentiation of users and non-users is pivotal to the relation modelling in the PORTP method. The exploitation of distinguished trajectory features is important to the intent classification as well as the STP models. It can be seen from Figure 7 and Figure 8 that such distinguished features are visible in the escalator and information kiosk scenes. The non-users appear to form a massive flow that moves past the objects at some distance. The users in the escalator scene preferred to select the four routes that converge before entering the engaged locations of the object. The users of the information kiosk demonstrated unique patterns, including stopping and turning. However, the trajectories of the users and the non-users of the ticket office are hardly differentiable.

When the scenes are examined more closely, there appears to exist a certain critical perimeter around every functional object. Inside the perimeter, the trajectories between users and non-users are different; outside the perimeter, the users and non-users are very similar in how they move. For example, the perimeter of divergence lies around 3 m to 5 m from the object for the information kiosk and the escalator scenes. However, the ticket office’s perimeter is exceptionally close to the object. In other words, there are hardly any differences between the users and non-users in their entire trajectories, except for the last meter.

As PORTP was designed to exploit features that differentiate different relation types, such as the users and non-users of an object, the method is more suitable for the information kiosk and escalator scene than the ticket office scene. The experiment results of the intent classifiers and the ADE and FDE of trajectory predictions were found to support this hypothesis.

In the training of the intent classifier, the use of the positional ground truth is based on the above observation that the intent is often uncertain, and the degree of uncertainty depends on the location. For example, the intent is often most uncertain when the pedestrian is outside the perimeter of divergence. The pedestrian ground truth is derived from the evidence of engaging with the functional object in one part of the original trajectory. However, other parts in the trajectory often lack the features that substantiate the pedestrian ground truth. Therefore, the models trained with positional ground truth performed significantly better than those trained with pedestrian ground truth.

Any feature in the trajectory that represents the intent to engage a function object may emerge at a long distance. As illustrated in Figure 9, there is a long trajectory between the information kiosk and the location where the pedestrian turned towards the functional object. The data-driven nature of PORTP is dependent on a training dataset that has recorded rich pedestrian–object interactions and long observations of pedestrian movement. Among the publicly available datasets, the ATC dataset [51] adopted in the experiment can satisfy the requirement. The datasets of short observation spans are likely to have missed the critical features in the trajectories for the model training. The long-term tracking of a large number of pedestrians is costly and technically challenging, but such resources are imperative for further research in this topic. 

## 6. Conclusions

This study represents one of the first attempts at including functional objects in pedestrian trajectory modelling. It suggests a definition of functional objects, and the relation between pedestrians and objects explains the heterogeneous movement patterns observed near functional objects in urban scenes. A method of tracking pedestrian–object relation and, based on the relation, using specialized trajectory prediction models has been presented. The results of the experiments indicate that the prediction models specifically trained for functional object features are generally better than those that do not. 

The findings of the experiments offer a reasonable baseline for future work. Pedestrians’ intent is often uncertain, especially when they are some distance from a nearby functional object. Furthermore, the intent often changes while moving [50], or their mind is not made up. To improve the accuracy of the intent classifier, a model or a class for uncertain pedestrians can be considered. In addition, the trajectories of the uncertain pedestrians may be annotated and used for training a STP model. 

## Figures and Tables

**Table 1:** Key statistics of the raw and the training dataset.

	Information Kiosk	Ticket Office	Escalator
Original Dataset			
Approximated Size of the ZOC	59.25 m^2^	294.00 m^2^	400.00 m^2^
Average Trajectory Length (SD)	101.6 s (21.7 s)	191.2 s (39.2 s)	66.9 s (38.9 s)
Total # Trajectories/# Pedestrians	1188	9357	26,076
Annotated *user*	1789	70	980
Annotated *non-user*	10,092	9287	25,096
Average Speed of *user*	0.834 m/s	0.937 m/s	0.783 m/s
Average Speed of *non-user*	1.089 m/s	0.916 m/s	0.968 m/s
After Resampling and Annotation			
Training Sample #	442,575	1,161,550	993,464
Annotated *user*	63,398	55,969	14,721
Annotated *non-user*	379,177	1,105,581	978,743

**Table 2:** Performance comparison of PORTP, the baselines GRU only and PoPPL.

			Information Kiosk	Ticket Office	Escalator
Whole Set	GRU Only	FDE (m)	0.544	0.456	0.660
		ADE (m)	0.359	0.323	0.414
	PoPPL	FDE (m)	0.555	0.456	0.670
		ADE (m)	0.369	0.326	0.410
	PORTP	FDE (m)	0.538	0.458	0.637
		ADE (m)	0.362	0.335	0.393
Only Users	GRU Only	FDE (m)	0.665	0.486	0.850
		ADE (m)	0.423	0.340	0.512
	PoPPL	FDE (m)	0.685	0.425	0.633
		ADE (m)	0.439	0.313	0.389
	PORTP	FDE (m)	0.644	0.398	0.591
		ADE (m)	0.420	0.294	0.373
Within 3 m of the Functional Object	GRU Only	FDE (m)	0.644	0.544	0.801
		ADE (m)	0.426	0.355	0.489
	PoPPL	FDE (m)	0.661	0.541	0.825
		ADE (m)	0.440	0.361	0.485
	PORTP	FDE (m)	0.640	0.543	0.785
		ADE (m)	0.429	0.368	0.468

**Table 3:** Performance comparison of various architectures for training the intent classifier.

			Information Kiosk		Ticket Office		Escalator	
PoPPL	Recall		0.723		0.758		0.480	
	Precision		0.726		0.616		0.734	
	F1		0.725		0.680		0.581	
	TN	FN	13,692	570	59,248	570	38,405	1311
	TP	FP	1487	561	1785	1111	1211	439
PORTP	Recall		0.761		0.782		0.627	
	Precision		0.813		0.777		0.809	
	F1		0.786		0.780		0.707	
	TN	FN	13,739	523	59,191	627	38,922	794
	TP	FP	1665	383	2250	646	1335	315

**Table 4:** A comparison between the two targets of locational ground truth and pedestrian ground truth for training the intent classifier.

			Information Kiosk		Ticket Office		Escalator	
PORTP (locational ground truth, probability distribution)	Recall		0.761		0.782		0.627	
	Precision		0.813		0.777		0.809	
	F1		0.786		0.780		0.707	
	TN	FN	13,739	523	59,191	627	38,922	794
	TP	FP	1665	383	2250	646	1335	315
PORTP (pedestrian ground truth, binary)	Recall		0.701		0.439		0.312	
	Precision		0.780		0.297		0.878	
	F1		0.738		0.355		0.460	
	TN	FN	13,580	682	58,717	1101	36,518	3198
	TP	FP	1598	450	861	2035	1448	202

**Table 5:** The performance of the intent classifier (the full architecture of PORTP and locational based ground-truth) predicts cases at different distances from the functional objects.

			Information Kiosk		Ticket Office		Escalator	
Far-range (>6 m)	Recall		0.785		0.610		0.545	
	Precision		0.757		0.574		0.725	
	F1		0.771		0.591		0.622	
	TN	FN	4776	29	42,367	389	30,528	553
	TP	FP	106	34	608	451	662	251
Mid-range (3 m to 6 m)	Recall		0.795		0.677		0.619	
	Precision		0.755		0.748		0.873	
	F1		0.774		0.711		0.724	
	TN	FN	7201	193	12,167	183	5723	186
	TP	FP	746	242	383	129	302	44
Near-range (<3 m)	Recall		0.730		0.887		0.831	
	Precision		0.884		0.879		0.933	
	F1		0.799		0.883		0.880	
	TN	FN	1762	301	4564	148	2652	74
	TP	FP	813	107	1164	161	365	26

**Table 6:** Performance comparison of the STP models.

		Information Kiosk	Ticket Office	Escalator
The *non-user, passing-by* model (GRU-Only)	FDE (m)	0.520	0.455	0.691
	ADE (m)	0.349	0.324	0.454
The *non-user, passing-by* model (PORTP)	FDE (m)	0.509	0.459	0.674
	ADE (m)	0.347	0.335	0.424
The *user, approaching* model (GRU-Only)	FDE (m)	0.547	0.331	0.404
	ADE (m)	0.374	0.273	0.290
The *user, approaching* model (PORTP)	FDE (m)	0.522	0.305	0.395
	ADE (m)	0.363	0.252	0.285

## Data Availability

The dataset used in the experiment can be downloaded from https://dil.atr.jp/crest2010_HRI/ATC_dataset, accessed on 30 March 2021.
